# Crop performance and profitability for the initial transition years of a regenerative cropping system in the Upper Midwest United States

**DOI:** 10.1002/jeq2.70084

**Published:** 2025-09-23

**Authors:** Ashim Datta, Brook Wilke, Christine Charles, Marc Hasenick, Tayler Ulbrich, Maninder Singh, Molly Sears, G. Philip Robertson

**Affiliations:** ^1^ W. K. Kellogg Biological Station Michigan State University Hickory Corners Michigan USA; ^2^ Department of Plant, Soil and Microbial Sciences Michigan State University East Lansing Michigan USA; ^3^ Great Lakes Bioenergy Research Center Michigan State University East Lansing Michigan USA; ^4^ Division of Soil and Crop Management ICAR‐Central Soil Salinity Research Institute Karnal India; ^5^ Hasenick Farm Springport Michigan USA; ^6^ Department of Agricultural, Food, and Resource Economics Michigan State University East Lansing Michigan USA

## Abstract

The transition from conventional to more regenerative cropping systems can be economically risky due to variable transition period yields and unforeseen costs. We compared yields and economic returns for the first 3 years of the transition from a business as usual (BAU) conventional corn (*Zea mays*)–soybean (*Glycine max*) rotation to an aspirational (ASP) five‐crop (corn‐soybean‐winter wheat [*Triticum aestivum*]–winter canola [*Brassica napus*]‐forage) rotation in the Upper Midwest United States. Regenerative ASP cropping practices included the more diverse crop rotation, continuous no‐till, cover crops, precision inputs, and livestock (compost) integration. For the first two transition years, BAU corn yields were 8%–12% higher than ASP while in the third transition year, BAU corn yields were 5% lower. Soybean yields were similar for the first 2 years but higher in BAU in the third year due to an ASP pest outbreak. Equivalent yields for other ASP crops were lower than BAU in the first 2 years but similar in the third year except for canola, which suffered from slug damage. Whole‐system economic returns narrowed across years; by year three, whole system comparisons for the ASP corn and soybean entry points (corn‐soybean‐wheat and soybean‐wheat‐canola, respectively) showed equivalent economic returns for BAU and ASP, despite yield differences, owing largely to the ASP system's reduced operational costs. Overall findings suggest that early regenerative systems can be as profitable as conventional systems with careful attention to rotation entry points and inputs.

## Core Ideas

- During the transition from conventional to regenerative agriculture, yields and profits can be variable.
- We tracked yields and economic returns for the first 3 years of a regenerative system in the Upper Midwest United States.
- Regenerative crop yields were sometimes lower than conventional yields, but the gap closed over the course of the study.
- Overall economic returns were equivalent for similar entry points because of lower regenerative operation costs.
- Economic risks of transitioning to regenerative practices can be minimized with careful attention to entry point.

## Plain Language Summary

Regenerative agriculture typically involves crop diversification, year‐round plant cover, and tillage reduction. There are many benefits, but the transition from conventional agricultural practices can be economically risky. We provide an economic analysis for the first 3 years of a long‐term regenerative agriculture experiment in the Upper Midwest United States to quantify overall returns for a system with all practices combined. Compared to a conventional corn–soybean rotation, crops in the more complex regenerative system exhibited somewhat lower yields but with differences closing over the course of 3 years. In contrast, economic returns were equivalent for specific entry points owing mostly to lower operational costs for the regenerative system. We conclude that careful attention to initial crop choice and agronomic inputs can readily mitigate the economic risk of transitioning to regenerative agricultural practices.

**Abbreviations**
ACSE: Aspirational Cropping System Experiment
ASP: aspirational
B:C ratio: benefit to cost ratio
BAU: business as usual
KBS: Kellogg Biological Station
LTAR: Long‐Term Agroecosystem Research
SEY: soybean equivalent yields

## 1 INTRODUCTION

The Midwestern United States is one of the most intensive agricultural areas in the world, producing over 33% of the world's corn (*Zea mays*) and 34% of the world's soybeans (*Glycine max*), and plays an important role in the US food system (Wang et al., 2020). Its sustained productivity through improved agronomic practices is important for both food security and farm profitability. Yet, future progress is challenged by climate change and other disrupters, and there is a crucial need to develop agronomic management systems that are resilient to these changes, with high yields, stable profits, and improved environmental outcomes.

Diversifying corn–soybean rotations with greater rotational complexity and cover crops is a potential strategy for providing resilience (Bowles et al., 2020; Bybee‐Finley et al., 2024). Yang et al. (2024), for example, showed that diversifying a wheat–maize cereal system in the North China Plain with legumes and cash crops enhanced equivalent yields by 38% over 6 years. Volsi et al. (2022) reported that crop diversification improved productivity and profitability in southern Brazil by 37% on average over 5 years, compared to conventional corn–soybean systems. Chahal et al. (2020) reported that cover crops increased crop yield, reduced yield variability, and provided greater profits for a temperate humid climate site in Ontario, Canada, studied for 9 years. In a global meta‐analysis, Vendig et al. (2023) reported that cover cropping simultaneously increased yields in 60% of 434 paired observations.

That said, while yield and other outcomes have been well documented, few authors other than LaCanne and Lundgren (2018) have attempted to assess the field‐scale economics of regenerative farming systems in which different conservation practices are stacked together, and fewer still during the transition from conventional farming. There is thus a large knowledge gap regarding the economic trajectory and consequences of adopting regenerative cropping.

As defined here, regenerative systems (sensu Giller et al., 2021) are those that incorporate the combined effects of greater crop diversity, winter cover (year‐round living roots), and little if any tillage in order to build soil health. Putatively, these systems better deliver ecosystem services related to water quality, biodiversity conservation, greenhouse gas mitigation, and more resilient yields and economic returns, compared to adjacent conventional systems as typified by corn–soybean agriculture in the Midwest United States. Yet, while many authors have noted the centrality of economics to the adoption of regenerative practices (e.g., Giller et al., 2021) and the need for economic data from long‐term trials with multiple practices (e.g., Khangura et al., 2023), economics data from even short‐term experiments are presently lacking.

Here, we describe productivity and economic returns for the transitional years of a recently established regenerative cropping systems experiment designed to contrast the resilience, profitability, and environmental performance of an advanced diversified cropping system (termed aspirational or ASP system, designed by farmers and agronomists) against a conventional corn–soybean system typical of the Upper Midwest United States (termed business as usual or BAU system). We contrast yields and net economic returns for a corn, soybean, winter wheat (*Triticum aestivum*), winter canola (*Brassica napus*), and perennial forage rotation, which also includes continuous no‐till, cover crops, precision inputs, and livestock integration (compost), against a corn and soybean rotation managed with prevailing practices for the region (Guo et al., 2023; Robertson et al., 2024).

Our specific objectives are to (i) evaluate crop yields for different BAU and ASP practices and (ii) compare net profits for the systems over the first 3 years of adoption. Initial results inform what farmers might expect upon first transitioning to regenerative practices, set the stage for the mid‐term trajectory, and identify early challenges to the later success of regenerative farming in the region.

## 2 MATERIALS AND METHODS

### 2.1 Experimental site

Our study was conducted in the northeastern part of the US Corn Belt at the Kellogg Biological Station (KBS) Long‐Term Agroecosystem Research (LTAR) site (ltar.kbs.msu.edu), located in southwest Michigan (42.3956 °N, 85.3749 °W; 288 m above sea level) (Robertson et al., 2024). The climate of the region is humid, continental, and temperate, with 30‐year average annual precipitation (1991–2020) of 926 mm year^−1^ (Hsieh et al., in press); about 1.3 m of snow falls per year (National Climatic Data Center, 2013). Precipitation throughout the year is evenly distributed although winter precipitation is somewhat lower (17% of total) compared to other seasons (26%–30%). Growing season precipitation (May through September) averages 417 mm year^−1^. Potential evapotranspiration exceeds precipitation in June, July, and August (Hamilton, 2015) and annually returns ∼59% of precipitation to the atmosphere (Hamilton et al., 2018). Mean annual temperature is 9.2°C, with monthly means (1991–2000) ranging from −4.4°C in January to 21.8°C in July (Hsieh et al., in press). Both mean annual temperature and precipitation have increased in recent decades; the average growing season length is about 2 weeks longer than in 1979 (Baffaut et al., 2025). Soil details appear in the Supporting Information.

### 2.2 Experimental details and management

The Aspirational Cropping System Experiment (ACSE) is part of the LTAR Network's Common Experiment (Liebig et al., 2024) and contrasts a corn–soybean system managed with regional prevailing practices (the Business as usual or BAU treatment) against a more complex ASP treatment. The ASP system is designed to deliver an enhanced suite of ecosystem services in addition to high and stable productivity (Kleinman et al., 2018; Spiegal et al., 2018).

**Core Ideas**

- During the transition from conventional to regenerative agriculture, yields and profits can be variable.
- We tracked yields and economic returns for the first 3 years of a regenerative system in the Upper Midwest United States.
- Regenerative crop yields were sometimes lower than conventional yields, but the gap closed over the course of the study.
- Overall economic returns were equivalent for similar entry points because of lower regenerative operation costs.
- Economic risks of transitioning to regenerative practices can be minimized with careful attention to entry point.

The BAU system is based on regional prevailing practices as determined by farm surveys and United States Department of Agriculture (USDA) statistics (Guo et al., 2023). A 2‐year corn–soybean rotation is chisel‐plowed in the fall or spring followed by secondary tillage pre‐plant. During corn years, N fertilizer (granular urea, 46% N and ammonium sulfate, 21% N) is spread in the spring (67 kg N ha^−1^) followed by liquid N (urea‐ammonium‐nitrate, 28% N) injected at planting (34 kg N ha^−1^) and again after corn emergence (123 kg N ha^−1^), for a total N application rate of 224 kg N ha^−1^). There are no cover crops in the BAU system (Tables S1–S3).

**FIGURE 1:**
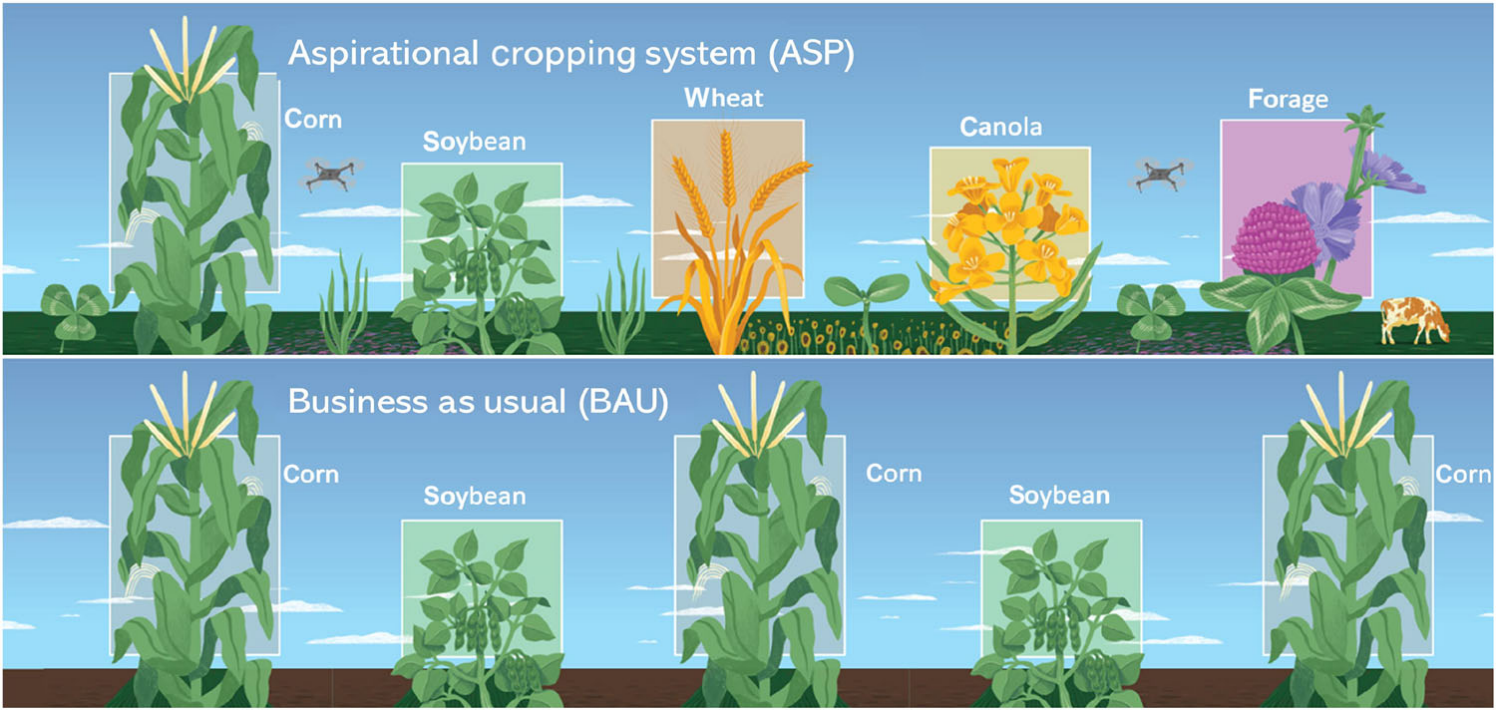
Conceptual model of the Kellogg Biological Station (KBS) Long‐Term Agroecosystem Research (LTAR) Aspirational Cropping Systems Experiment (ACSE). From Robertson et al. (2024).

The ASP system was co‐designed by stakeholders and researchers in a series of workshops initiated in 2021 (Guo et al., 2025) and consists of a five‐crop rotation in the sequence corn, soybean, winter wheat, winter canola (*B. napus*), and a forage mix consisting of alfalfa (*Medicago sativa*), red clover (*Trifolium pratense*), chicory (*Cichorium intybus*), and annual ryegrass (*Lolium multiflorum*) harvested for off‐site livestock consumption (Figure 1). This sequence of spring‐planted, fall planted, and perennial crops allows for optimal integration of cover crops, planted after corn and winter wheat. Crimson clover (*Trifolium incarnatum*), dwarf essex rapeseed (*B. napus*), and radish (*Raphanus raphanistrum*) are together interseeded into corn followed by cereal rye (*Secale cereale*) after harvest. Cover crops harvested for forage following winter wheat include a mixture of sorghum sudan grass (sudex; *Sorghum bicolor × drummondii*), pearl millet (*Pennisetum glaucum*), and sunn hemp (*Crotalaria juncea*). All phases of the rotation are managed with continuous (permanent) no‐till, precision fertilizer inputs, and integrated pest management. Manure is added prior to corn together with synthetic N, which is also added to other crops except soybean at rates that range from 34 to 179 kg N ha^−1^ year (Tables S1–S3).

The KBS ACSE is a randomized complete block design experiment, with all rotation phases presenting each year, as detailed in Robertson et al. (2024) (Figure S1). The two treatments are replicated in four blocks, resulting in eight BAU plots (2 rotation phases × 4 blocks) and 20 ASP plots (5 rotation phases × 4 blocks) for a total of 28 plots, each 28 m × 85 m (0.24 ha).

The experiment was initiated in 2022. Prior to the start of the experiment, corn, soybean, and wheat were grown from 2016 to 2021 using prevailing practices, concluding with a corn grain crop in 2021 that served as a pre‐treatment baseline for the experiment. Because the pre‐treatment corn crop was not fully harvested until November, 2021, spring rather than fall varieties of wheat, canola and perennial forages were planted in the first year of the experiment (early spring 2022), despite the expectation of lower yields compared to fall planted crops.

Specific treatment names with corresponding crops for the first 3 years of the experiment are presented in Table 1. Individual crop management practices for each year appear in Tables S1–S3.

**TABLE 1:** Crop rotation phases by year; cover crops not shown.

System	Rotation phase	2022	2023	2024
Business as usual	1	Corn	Soybean	Corn
	2	Soybean	Corn	Soybean
Aspirational	1	Corn	Soybean	Wheat
	2	Soybean	Wheat	Canola
	3	Wheat^a^	Canola	Perennial forage
	4	Canola^a^	Perennial forage	Corn
	5	Perennial forage^a^	Corn	Soybean

*Note*: See narrative for further descriptions.

^a^ Spring rather than fall planted (2022 only).

### 2.3 Crop yields

Crops were harvested with commercial scale equipment, and yields were measured with weigh wagons. Grain yields are expressed on a 15.5% moisture basis for corn, 13% for soybean, 13.5% for wheat, and 10% for canola. In addition to absolute yields, we also calculated SEY (Gathala et al., 2013) for corn, wheat, and canola grain and forage biomass in order to provide a common metric for comparing different crops with one another:

(1) $$\mathrm{SEY}=\frac{\mathrm{Crop}\;\mathrm{yield}\;\left(\mathrm{Mg}/\mathrm{ha}\right)\times \;\mathrm{Crop}\;\mathrm{price}\;\left(\$\mathrm{US}/\mathrm{Mg}\right)}{\mathrm{Price}\;\mathrm{of}\;\mathrm{soybean}\;\left(\$\mathrm{US}/\mathrm{Mg}\right)},$$

where crop yield and price refer to corn, wheat, canola, or forage biomass.

### 2.4 Economic analysis

Our economic analysis considers all production costs excluding land rent or ownership costs (Table 2). Variable costs include human labor, tractor use, the cost of production (tillage, planting, seeds, fertilizers, pesticides, spraying, harvesting, drying), and transport to market. Fixed costs include depreciation of machinery and interest on working capital. The cost of human labor is based on Michigan State University Extension rates for skilled and unskilled labor (Miller et al., 2023). Gross returns (GR) were calculated by multiplying yields of each crop by prices summarized by the USDA Risk Management Agency (USDA Risk Management Agency (RMA), 2024) for a given year, using the agency's Price Discovery Tool which is used to generate values for crop insurance purposes. Forage and straw values were calculated using current local market rates. Net returns were calculated as the difference between gross returns and total cost. The benefit:cost ratio (B:C ratio) was calculated by dividing gross income by total costs. All economic data are in US dollars for the reporting year.

**TABLE 2:** Production costs and sale prices for each system and rotation phase. Note that values in first column of Sales rice are in Imperial not SI units.

				Production Cost		Sales price		
Year	System	Rotation phase	Crop	$ acre^−1^	$ ha^−1^	$	Unit (Imperial)	$ Mg^−1^
2022	BAU	1	Corn	758	1872	6.68	bu	263
		2	Soybean	416	1028	14.43	bu	530
	ASP	1	Corn	627	1549	6.68	bu	263
		2	Soybean	293	725	14.43	bu	530
		3	Wheat (spring)	318	785	8.12	bu	320
			Cover crop (forage)	224	554	150	dry ton	165
		4	Canola (spring)	331	818	0.37	lb	817
		5	Perennial forage	456	1127	200	dry ton	221
2023	BAU	1	Soybean	372	920	13.76	bu	542
		2	Corn	779	1924	5.91	bu	233
	ASP	1	Soybean	380	938	13.76	bu	542
		2	Wheat grain	364	900	8.45	bu	333
			Wheat straw	57	141	120	dry ton	132
			Cover crop (forage)	207	512	150	dry ton	165
		3	Canola	384	948	0.31	lb	684
		4	Perennial forage	471	1163	200	dry ton	221
		5	Corn	639	1578	5.91	bu	233
2024	BAU	1	Corn	721	1781	4.66	bu	184
		2	Soybean	422	1042	11.55	bu	425
	ASP	1	Wheat	415	1025	6.72	bu	247
			Straw	48	119	120	dry ton	132
			Cover Crop (forage)	214	529	160	dry ton	176
		2	Canola	357	882	0.27	lb	596
		3	Perennial forage	533	1317	160	dry ton	176
		4	Corn	674	1666	4.66	bu	184
		5	Soybean	462	1141	11.55	bu	425

Abbreviations: ASP, aspirational; BAU, business as usual.

### 2.5 Statistical analysis

Data were subjected to analysis of variance (ANOVA) using the general linear model procedure of the SPSS Windows version 16.0 (SPSS Inc., Chicago, USA). Treatment means were separated by Duncan multiple range test at a 5% level of significance (*p* < 0.05) (Gomez & Gomez, 1984).

## 3 RESULTS

### 3.1 Growing conditions

In 2022, total and growing season (May–September) rainfall was 851 and 429 mm, respectively, or 8% below and 2% above normals (see Section 2.1). The average temperature for the growing season was 19.7°C with mean maximum and minimum temperatures of 25.7 and 13.7°C, respectively (Figure 2a). In 2023, total and growing season rainfall was 950 and 307 mm, respectively, or 2% above and 26% below normals. The average temperature for the growing season was 18.9°C with mean maximum and minimum temperatures of 25.5°C and 12.2°C, respectively (Figure S2b). In 2024, total and growing season rainfall was 1062 and 510 mm, respectively, or 14% and 22% above normal. The average temperature for the growing season was 20.0°C, with mean maximum and minimum temperatures of 26.5 and 13.8°C, respectively (Figure S2c).

### 3.2 Crop yields

#### 3.2.1 Crop yields

Across all years and treatments, corn yields varied from 8.62 to 12.0 Mg ha^−1^, soybean yields from 2.80 to 4.53 Mg ha^−1^, winter wheat (grain only) from 3.77 to 5.55 Mg ha^−1^, winter canola from 1.41 to 2.37 Mg ha^−1^, and forage from 4.67 to 14.0 Mg ha^−1^ (Table 3).

**TABLE 3:** Absolute and soybean equivalent yields for 2022–2024.

	Absolute yields (Mg ha^−1^)			Soybean‐equivalent yields (Mg ha^−1^)		
System	2022	2023	2024	2022	2023	2024
Business as usual						
Corn	11.51 ± 0.38a	9.83 ± 0.16a^a^	11.48 ± 0.42b	5.31 ± 0.18a	4.50 ± 0.17a	5.11 ± 0.19a
Soybean	4.44 ± 0.01 cd	3.07 ± 0.13ef	4.53 ± 0.04d	4.44 ± 0.01c	3.07 ± 0.13b	4.53 ± 0.04b
Aspirational						
Corn	10.61 ± 0.30b^a^	8.62 ± 0.21b	12.00 ± 0.33b	4.90 ± 0.12b	4.08 ± 0.10a	5.34 ± 0.15a
Soybean	4.07 ± 0.09d	2.80 ± 0.15f	3.50 ± 0.16e	4.07 ± 0.09c	2.80 ± 0.15b	3.50 ± 0.16c
Wheat grain	2.42 ± 0.17e^b^	3.77 ± 0.13d	5.55 ± 0.20c	2.06 ± 0.14d^b^	4.06 ± 0.13a^c^	5.21 ± 0.17a^c^
Wheat straw		3.16 ± 0.10ef	2.15 ± 0.11f			
Cover crop	2.04 ± 0.16ef	3.54 ± 0.19de	3.82 ± 0.13de			
Canola	1.20 ± 0.02f^b^	1.41 ± 0.23 g	2.37 ± 0.04f	1.70 ± 0.03d^b^	1.84 ± 0.30c	3.22 ± 0.06c
Perennial forage	5.05 ± 0.68c	4.67 ± 0.16c	14.02 ± 0.60a	1.24 ± 0.24e	1.77 ± 0.06c	5.06 ± 0.22a

*Note*: Values are mean ± SE (*n* = 4). Means within a column followed by the same letter are not different at *p* = 0.05.

^a^ *n* = 3 because of severe wildlife damage in one plot.

^b^ Spring rather than fall for 2022 only.

^c^ Includes straw and cover crop yields.

In 2022, BAU corn yields (11.5 Mg ha^−1^) were 7.8% higher than ASP corn yields (10.6 Mg ha^−1^). BAU and ASP soybean yields (4.44 and 4.07 Mg ha^−1^, respectively) were statistically similar. The ASP forage crop in 2022 yielded 5.05 Mg ha^−1^, spring wheat 2.42 Mg ha^−1^, and spring canola 1.20 Mg ha^−1^ (Table 3). In 2023, BAU corn yields were again significantly higher than ASP (9.83 vs. 8.62 Mg ha^−1^), while soybean yields were statistically similar (3.07 and 2.80 Mg ha^−1^, respectively). ASP winter wheat yields in 2023 were 3.77 Mg ha^−1^, forage 4.67 Mg ha^−1^, and winter canola low (1.41 Mg ha^−1^) due mainly to slug damage. In 2024, ASP corn yields (12.0 Mg ha^−1^) were numerically 4.5% higher than BAU corn yields (11.5 Mg ha^−1^), though statistically similar. BAU soybean yields in 2024 (4.53 Mg ha^−1^) were 23% higher than ASP soybean yields (3.50 Mg ha^−1^), which suffered from slug damage in 2024. The 2024 ASP forage crop yielded 14.0 Mg ha^−1^, winter wheat 5.55 Mg ha^−1^, and winter canola 2.37 Mg ha^−1^.

#### 3.2.2 Soybean equivalent yields

BAU corn exhibited the highest SEY in 2022 (5.31 Mg ha^−1^), numerically but not significantly higher than ASP corn (4.90 Mg ha^−1^) (Table 3). The SEY of BAU soybean (4.44 Mg ha^−1^) was numerically higher than that of ASP soybean (4.07 Mg ha^−1^), and the SEY of ASP spring wheat was 2.06 Mg ha^−1^, spring canola 1.70 Mg ha^−1^, and forage 1.24 Mg ha^−1^—53%, 61%, and 72% lower than BAU soybean, respectively. In 2023, the SEY of BAU corn (4.50 Mg ha^−1^) was 9% higher than ASP corn (4.08 Mg ha^−1^) compared to BAU and ASP soybean (3.07 and 2.80 Mg ha^−1^). SEY for ASP wheat (4.06 Mg ha^−1^) was similar to that for ASP corn, whereas winter canola (1.84 Mg ha^−1^) and forage (1.77 Mg ha^−1^) SEY were 40% and 42% lower than BAU soybean, respectively. In 2024, the SEYs for BAU corn (5.11 Mg ha^−1^), ASP corn (5.34 Mg ha^−1^), ASP wheat (5.21 Mg ha^−1^), and ASP forage (5.06 Mg ha^−1^) were statistically similar. The SEYs for ASP soybean and ASP canola were 23% and 29% lower than BAU soybean, respectively, whereas the SEY for ASP forage was 11.7% higher than for BAU soybean.

For the combined years, BAU corn–soybean–corn SEY (4.50 Mg ha^−1^) was 8.4% higher than ASP corn–soybean–wheat SEY (4.12 Mg ha^−1^). BAU soybean–corn–soybean SEY (4.49 Mg ha^−1^) was numerically 15.8% higher than ASP soybean–wheat + cover crop–canola SEY (3.78 Mg ha^−1^), though statistically similar. BAU corn–soybean–corn SEY (4.50 Mg ha^−1^) and ASP soybean–wheat + cover crop–canola SEY (3.78 Mg ha^−1^) were also similar, whereas the SEY of wheat + cover crop‐canola‐forage was 3.16 Mg ha^−1^, canola‐forage‐corn was 2.94 Mg ha^−1^, and forage‐corn‐soybean was 3.26 Mg ha^−1^ (calculated from values in Table 3).

### 3.3 Economics

#### 3.3.1 Cost of production

In 2022, the costs of the production of BAU corn and soybean were 17% and 27% higher than for ASP corn and soybean, respectively (Table 2). In 2023, the cost of production for BAU corn was again higher than ASP corn, but ASP and BAU soybean costs were similar due to the addition of an ASP rye cover crop and manure costs. In 2024, the cost of production of BAU corn was again higher than ASP corn, but ASP soybean costs were 10% higher than BAU costs because of the need to replant ASP soybean following a slug outbreak.

The costs of the production of ASP canola ranged from $818 to 948 ha^−1^ over the 3 years, for wheat (including grain, straw, and subsequent cover crop harvested for forage), from $1339 to 1673 ha^−1^, and for forage, from $1127 to 1317 ha^−1^.

Across all 3 years (Table 4), most ASP systems had lower production costs than BAU. BAU corn–soybean–corn systems had a 6% higher cost of production than ASP corn–soybean–wheat systems, and BAU soybean–corn–soybean systems were 20% costlier than ASP soybean‐wheat‐canola systems. BAU systems were also 16%–21% costlier than ASP wheat–canola–forage, canola–forage–corn, and forage–corn–soybean systems.

**TABLE 4:** Mean economic performance across all years (2022, 2023, 2024) for cropping systems with corn or soybean entry points.

System	Total cost ($ ha^−1^)	Gross return ($ ha^−1^)	Net return ($ ha^−1^)	B:C ratio
BAU				
Corn–soybean–corn	1528	2619a	1091a	1.75b
Soybean–corn–soybean	1329	2514a	1185a	2.05a
ASP				
Corn–soybean–wheat	1435	2571a	1057a	1.79b
Soybean–wheat–canola	1067	2137b	1070a	2.18a
Wheat–canola–forage	1201	1668c	467b	1.40c
Canola–forage–corn	1216	1571c	600b	1.44c
Forage–corn–soybean	1282	1732c	450b	1.33c

*Note*: Means within a column followed by the same letter are not different at *p* = 0.05. Values are means ± SE (*n* = 3 years).

Abbreviations: ASP, aspirational; BAU, business as usual; B:C, benefit:cost ratio.

#### 3.3.2 Profitability

##### 3.3.2.1 Economic performance in 2022

Net returns and B:C ratios are presented in Table 5. In 2022, as noted earlier, ASP systems had lower production costs than BAU systems. However, BAU systems achieved higher yields and therefore higher gross returns, resulting in numerically higher net returns for BAU corn ($1693 ha^−1^) compared to ASP corn ($1603 ha^−1^), and more similar net returns for soybeans ($1715 BAU vs. 1689 ha^−1^ ASP).

**TABLE 5:** Economic analysis for different cropping systems.

	2022				2023				2024			
Crop	Total cost ($ ha^−1^)	Gross return ($ ha^−1^)	Net return ($ ha^−1^)	B:C ratio	Total cost ($ ha^−1^)	Gross return ($ ha^−1^)	Net return ($ ha^−1^)	B:C ratio	Total cost ($ ha^−1^)	Gross return ($ ha^−1^)	Net return ($ ha^−1^)	B:C ratio
**Business as usual system**												
Corn	1884	3577 ± 119a	1693 ± 119a	1.90 ± 0.06a	1924	2618 ± 97a	694 ± 97a	1.36 ± 0.05c	1780	2495 ± 92ab	715 ± 92bcd	1.40 ± 0.05c
Soybean	1022	2711 ± 5c	1689 ± 5a	2.65 ± 0.01b	920	1786 ± 74c	866 ± 74a	1.94 ± 0.08a	1042	2214 ± 20b	1172 ± 20a	2.12 ± 0.02a
Mean	1453	3144	1691	2.28	1422	2202	780	1.65	1411	2355	944	1.76
**Aspirational system**												
Corn	1695	3298 ± 81b	1603 ± 94a	1.95 ± 0.06c	1578	2376 ± 57b	798 ± 57a	1.51 ± 0.04c	1666	2608 ± 72a	942 ± 72ab	1.57 ± 0.04c
Soybean	767	2482 ± 56c	1715 ± 56a	3.21 ± 0.07a	938	1632 ± 88c	694 ± 88a	1.74 ± 0.09b	1141	1708 ± 76c	567 ± 76d	1.50 ± 0.07c
Wheat^a^	1339	1172 ± 79d	−167 ± 79c	0.88 ± 0.06e	1553	2360 ± 78b	807 ± 78a	1.52 ± 0.05c	1671	2784 ± 228a	874 ± 84bc	1.67 ± 0.14b
Canola	818	1077 ± 19d	259 ± 19b	1.32 ± 0.02d	948	1360 ± 112d	412 ± 112b	1.43 ± 0.12c	882	1571 ± 27c	689 ± 27 cd	1.78 ± 0.03b
Forage	1127	1113 ± 149d	−14 ± 149c	0.99 ± 0.13e	1163	1029 ± 34e	−134 ± 34.5c	0.88 ± 0.03d	1316	2473 ± 106ab	1157 ± 106a	1.88 ± 0.08b
Mean	1149	1825	679	1.67	1236	1751	515	1.42	1335	2229	846	1.68

*Note*: Wheat includes straw and cover crop forage sales. Values are means ± SE (*n* = 4). Means within a column followed by the same letter are not different at *p* = 0.05. For rotation sequence, see Table 2.

Abbreviations: ASP, aspirational; BAU, business as usual; B:C, benefit:cost ratio.

^a^ Includes harvested cover crop and straw except no straw in 2022.

Other ASP crops showed mixed results: spring‐planted canola had a modest net return ($259 ha^−1^), while spring‐planted wheat and spring‐planted forage both had negative net returns. The highest B:C ratio was observed for ASP soybean in 2022 at 3.21, about 17% higher than BAU soybean (2.65). Corn B:C ratios were similar across systems (1.95 ASP vs. 1.90 BAU).

##### 3.3.2.2 Economic performance in 2023

In 2023, ASP corn maintained an 18%–19% cost advantage over BAU corn, though corn yields and thus gross returns remained higher in the BAU system; soybean yields and gross returns were statistically similar between systems. Net returns ($ ha^−1^) for corn were consequently higher in the ASP system (798 ASP vs. 694 BAU) but lower for soybeans (694 ASP vs. 866 BAU). Notably, ASP corn had a higher B:C ratio than BAU corn (1.51 vs. 1.36, respectively), while the reverse was true for soybean (1.94 BAU vs. 1.74 ASP).

ASP forage had the lowest net returns in 2023 ($−134 ha^−1^), while net returns for wheat and canola were lower ($807 and 412 ha^−1^, respectively), with corresponding B:C ratios that were competitive (1.52 and 1.43) despite low canola yields due to a slug outbreak. Forage, on the other hand, was not competitive in 2023, with a B:C ratio <1.

##### 3.3.2.3 Economic performance in 2024

By 2024, ASP corn outperformed BAU corn in yield and thus gross returns; this, together with 6% lower production costs in ASP corn, led to 32% greater net returns for ASP corn ($942 vs. 715 ha^−1^). ASP soybean had somewhat higher production costs ($1141 ASP vs. 1042 BAU ha^−1^) owing to the need to replant due to slug damage, which also substantially impacted yields and thus net returns ($1172 ASP vs. 567 BAU ha^−1^).

ASP forage showed strong economic performance with net returns of $1157 ha^−1^, comparable to BAU soybean. Net returns for canola ($689 ha^−1^) were substantially greater than in 2023 ($412 ha^−1^), with a strong B:C ratio of 1.78. B:C ratios for other ASP crops ranged from 1.50 to 1.88, compared to B:C ratios for BAU corn and soybean of 1.40 and 2.12, respectively.

##### 3.3.2.4 Three‐year system averages

Three‐year BAU and ASP systems with the same corn or soybean entry points had statistically similar net returns that ranged from $1057 ha^−1^ for ASP corn—soybean‐wheat to $1185 ha^−1^ for BAU soybean–corn–soybean systems (Table 4). In contrast, ASP systems that started with spring‐planted wheat, canola, or forage lagged behind BAU by 45%–62% with returns that ranged from $450 to 600 ha^−1^.

Notably, B:C ratios were similar for BAU soybean–corn–soybean (2.05) and soybean–wheat–canola (2.18) systems, and likewise for BAU corn–soybean–corn (1.75) and ASP corn–soybean–wheat (1.79) systems. As for net returns, B:C ratios were substantially lower for ASP systems that started with spring‐planted wheat, canola, or forage (1.33–1.44).

Differences between BAU and ASP for both net returns and B:C ratios narrowed over the 3 years of the study (Table 5). In 2022, the mean net return for the BAU system across all crops ($1691 ha^−1^) was 2.5 times greater than that for the ASP system ($679 ha^−1^); by 2023 and 2024, the advantage had shrunk to 1.37 and then 1.11. Likewise, differences in average B:C ratios declined from an average BAU‐ASP difference of 0.61 in 2022 to 0.23 in 2023 to 0.08 in 2024, indicating economic convergence between the systems for at least these first 3 years.

## 4 DISCUSSION

All phases of each cropping system were profitable in all three transition years. That said, the BAU system was, on average, more profitable than the ASP, but over the 3‐year period, the difference diminished from 2.5 times more profitable in 2022 to 1.4 times in 2023 and to 1.1 times in 2024 (Table 5). However, when the ASP system started with either corn or soybean, net profits were equivalent between the two systems over the entire 3‐year period (Table 4): although BAU yields were usually (but not always) higher, ASP production costs were always lower, offsetting the greater BAU returns, and starting with corn or soybean avoids the opportunity cost of initiating the ASP rotation with lower yielding spring‐planted rather than fall‐planted wheat, canola, or forage.

### 4.1 Yield patterns

Corn yields in the BAU system were higher than in the ASP system for the first 2 years of the transition but similar for the third year. Soybean ASP yields were numerically lower but statistically similar to BAU yields in the first 2 years but statistically lower in the third year. Equivalent yields for the other ASP crops were quite low for the first transition year; for the second and third years, equivalent yields for winter wheat were statistically similar to those for BAU corn (when including wheat straw and harvested cover crop), and by year three, equivalent yields for canola and forage were higher than for BAU corn. Thus, by year three, ASP corn closed its initial yield gap with BAU corn, as did winter wheat, canola, and forage; ASP soybean yields remained variable, largely due to unexpected pest pressure (see below).

The lower yield of ASP corn compared to BAU corn for the first two years was likely due to the transition to no‐till, known to take several years (if not a decade) to consistently out‐yield conventional tillage in our soils (Cusser et al., 2020); by year three, we observed numerically higher but statistically similar corn yields in the ASP system. Exacerbating the transition challenge in 2023 was a dry spring, made worse in ASP by cover crop water use. The low equivalent yields for other ASP crops in year one is likely due to the out of sequence spring wheat, canola, and forage sowing due to the starting conditions of the experiment. The ASP system design calls for late summer/fall planting of these crops, which was not possible in the first year (2022) because of a preceding corn crop in 2021 that was not harvested until October–November, 2021, too late for fall planting. We thus had to plant spring varieties of wheat and canola, known to yield less, and forfeit fall growth of the forage crops. Farmers transitioning out of corn or soybeans would face the same trade‐off should they choose to plant a transition crop that is normally fall‐planted. High forage yields in 2024 resulted from more rain together with warmer temperatures (Figure S2c) and also, for the first cutting, the addition of volunteer canola in the perennial forage seed mixture.

Other studies have found similar transition patterns for cropping systems with advanced practices like diversified rotations, no‐till, and cover crops. No‐till implementation, for example, often results in a short‐term yield depression. Delgado et al. (2024) found that immediately after conversion to no‐till, yields were numerically lower than yields from conventional till, but after 5 years, the yield gap closed. Pedersen and Lauer (2003) found in a 5‐year corn–soybean experiment in Wisconsin that yields decreased by 5% in 2 of the first 5 years for corn, but not for soybean, for which no‐till yielded 6% more than conventional till on average. In a study of a silage corn–soybean rotation conducted in Quebec, Canada, Whalen et al. (2007) also found depressed yields during the transition phase for 3 (corn) and 2 (soybean) years over the first 5 years. Che et al. (2023), in contrast, found no‐till and conventional till yields in a corn–soybean–wheat system in Maryland, USA, were not significantly different in any of 24 years. At the KBS, a 29‐year study documented consistent yield benefits for no‐till but only after 13 years (Cusser et al., 2020). In a global meta‐analysis of 678 paired studies both short and long term, Pittelkow, Linquist, et al. (2015) found that no‐till yields declined for most rainfed crops during the first 1–2 years of implementation, and thereafter were similar to conventional yields for legume crops and slightly lower for cereal crops.

The favorable yields in our ASP systems following the first 2 years could be due to a range of improved management practices acting alone or in combination. Importantly, our ASP system includes cover crops as well as a more complex rotation than the BAU system. In a 10‐year rotation study in Minnesota, USA, Crookston et al. (1991) reported a yield advantage when either corn or soybean followed several years of the other crop grown continuously. Morrison et al. (2018) reported that wheat yields were 22% higher on average when grown under rotation compared to monoculture over all tillage–rotation combinations, corn grown under rotation yielded ∼8% higher when grown with tillage, and yields for soybean grown under rotation did not change. DeFelice et al. (2006) proposed that the validity of tillage and rotation results improve after several years of experimentation due to the time required for the development of soil tilth, porosity, drainage, and a stable microbial environment. We would add pest management to this list in light of the slug outbreak in our ASP soybean and canola rotations.

It is also possible that the positive trajectory of our ASP system is transitory. Many authors (notably Rasmussen et al. (1998), Robertson et al. (2008), and Kleinman et al. (2018)) have highlighted the need for long‐term (>20 year) experiments when assessing the sustainability of agricultural management practices. Thus, it is too early to make long‐term conclusions about ASP versus BAU yield trends, although the patterns even to date are relevant for the early transition years and may help to guide the adoption of regenerative practices elsewhere. This may be particularly the case when designing systems to avoid yield declines due to specific agronomic stresses like slugs in our ASP soybean and canola rotations.

### 4.2 Economic returns

Despite yields that were often lower in the ASP system, ASP crops were always profitable and often more so than crops in the BAU system. For corn and soybean, economic returns in the ASP system were higher than in the BAU system for both corn (6%–22% higher) and soybean (5%–16% higher) for 2 out of 3 years (Table 5). In the other year, ASP profits were 20% and 40% lower than BAU for corn and soybean, respectively. Higher profitability in the ASP system largely reflected a lower cost of production. Lower profitability in the ASP system reflected unexpected agronomic challenges; in 2024, for example, ASP soybean suffered from a major early‐season slug outbreak that forced replanting, with its associated economic cost, and depressed yields by 23% (Tables 2 and 4).

Total profitability across all 3 years is possible for rotations with similar entry points, that is, those rotations starting with either corn or soybean (Table 4). A comparison of rotations with small grain entry points would be biased by the absence of fall‐planted crops in 2022. For these starting points, net returns for the entire 3‐year period do not significantly differ: the BAU corn–soybean–corn rotation returned $956 ha^−1^, whereas the ASP corn–soybean–wheat rotation returned a statistically similar $977 ha^−1^; likewise, the BAU soybean–corn–soybean rotation returned $1177 ha^−1^ versus the corresponding ASP soybean–wheat–canola rotation's statistically similar $1078 ha^−1^.

Thus, farmers considering transitioning to a regenerative system similar to ASP with cover crops, no‐till, and a more complex rotation than a corn–soybean system should prepare for slightly reduced yields during transition years, but can maintain similar profitability by choosing optimal entry points for complex rotations (e.g., in our case avoiding a spring‐planted canola, wheat, or forage entry point) and reaping reduced production costs associated with no‐till and fewer inputs (Table 5). Eventually, the influence of regenerative practices on improved crop growth and yield in the ASP system may allow the ASP system to consistently outperform the BAU system economically and perhaps even agronomically. Growing input costs may enlarge any economic disparities, insofar as machinery, fuel, and fertilizer costs often increase with little warning, whereas other input costs (e.g., seeds and pesticides) are more similar across systems.

Lower costs of production have been noted by others studying no‐till, in particular. Sijtsma et al. (1998) determined that when costs for seed, fertilizer, and herbicide were similar, no‐till systems were less expensive to implement due to reduced tractor traffic and fuel consumption. Likewise, Che et al. (2023) found higher profits in no‐till over conventional tillage for corn, soybean, and wheat crops for the same reasons, and over 24 years observed that relative profitability of no‐till increased as the practice was used longer over time. In addition, Cusser et al. (2020) conducted a comprehensive relative profitability analysis at the KBS to suggest that 13 years after initial implementation of no‐till, the purchased equipment costs were fully recovered, providing a higher probability of increasingly greater profit with longer duration.

Cover crops may also provide positive net returns by reducing the amount of N fertilizer and herbicides needed. In the US Corn Belt, Pratt et al. (2014) estimate that cover crop costs ranged from $33 to $70 per acre, while economic benefits ranged from $37 to $78 per acre. Economic benefits include nutrient scavenging; increased soil organic matter; weed suppression; and reduced nitrate leaching, soil erosion, and soil compaction. Roth et al. (2018) found that a cereal rye and daikon radish cover crop mix can recover approximately 61% of cover crop implementation costs by improving N cycling and reducing soil erosion and N losses to subsurface drainage under corn and soybean systems. In water‐limited regions, Bergtold et al. (2017) estimated that growing cover crops in the fallow phase of a crop‐fallow system can provide positive net returns. Volsi et al. (2022) observed higher profitability with species diversification compared to a conventional corn–soybean rotation, and Garbelini et al. (2020), in a long‐term Brazilian study involving soybean, corn, wheat, and tropical forage grasses, found that the more diversified production systems provided higher profits.

We did not include in our analysis potential conservation payments for soil or N conservation, carbon sequestration, or other ecosystem service related to environmental performance (Robertson et al., 2014; Swinton et al., 2006). Payments for implementing no‐till, cover crops, or reduced N fertilizer use could significantly improve the ASP system's profitability, easing the transition years in particular.

### 4.3 Early lessons learned

Transitioning to regenerative agricultural practices can be challenging for farmers all over the world due to possible yield penalties, especially during the initial years of a transition. Understanding the early effects of such transitions is critical for any crop on which farmers rely for their income and is important for informing agroecological strategies to cope with the challenges of climate change and food insecurity. Moreover, knowing which combinations of regenerative practices best deliver stable yields and profitability would be particularly helpful.

There are few if any studies that report yields and economic returns for the transition years to fully regenerative agriculture. Studies for individual practice effects are more common, whether conservation tillage (e.g., Pittelkow, Liang, et al., 2015), cover crops (e.g., Bergtold et al., 2017), or diversified rotations (e.g., Bowles et al., 2020; Bybee‐Finley et al., 2024). What have we learned from integrated systems‐level experiments like the present study?

First, in the present study, we have learned that the initial transition year should be preceded by a crop that allows fall planting of cover crops or winter cereals, assuming they are part of the diversified system. Our inability to plant winter wheat, winter canola, and fall‐seeded forage crops because of a preceding corn crop strongly depressed first year yields and consequent profitability. In the absence of the potential for fall seeding, our findings suggest that the initial transition year should be a standard spring planted crop like corn or soybean rather than substituting a spring‐planted for fall‐planted variety of cereal, oil seed, or forage crop.

Second, across the 3‐year ASP transition period, we were able to maintain whole‐rotation profitability equivalent to that of the BAU system by reducing inputs, with only modest yield effects. No‐till provided well‐recognized savings by removing tillage passes and by providing presumed fertility benefits like added soil moisture prior to seasonal droughts. Our substitution of composted manure for synthetic N allowed us to reduce the system's dependence on commercial fertilizer, providing a greater degree of nutrient recycling. Integrated pest management with weekly scouting allowed us to apply management interventions like fungicides more judiciously and avoid the use of foliar insecticides.

Third, unpredicted pest problems specific to the ASP system can hinder early success. In our case, a slug outbreak in the second and third years of ASP implementation caused a need to replant canola in 2023 and soybean twice in 2024. We do not yet fully understand the reasons for the outbreak, but likely it is a rotation sequence effect—the preceding no‐till corn crop with high residue retention may provide overwinter and egg‐laying habitat absent in the BAU system. Continuous living cover may also be a factor. We expect that better residue control, more timely crop planting and cover crop termination, and/or changing the crop sequence will mitigate this problem in subsequent years.

Fourth, while not documented here, we are already observing significant soil health benefits in the ASP system. For example, at the field scale, there has been substantially less downslope sheet erosion in ASP fields; this was particularly evident following a 38‐mm (1.5 inch) midspring storm in 2024. Differential effects of episodic weather events like these will become increasingly important to the resilience of these systems as the changing climate continues to deliver more variable and extreme weather. Likewise, we have been able to substantially reduce N and phosphorus fertilizer applications in ASP corn and soybean relative to BAU crops, due to cover crop inclusion and a single composted manure application.

We do not yet know the environmental implications of our regenerative practices. Studies of soil health, water quality, greenhouse gas emissions, carbon sequestration, insect and microbial biodiversity, and other outcomes are underway and will provide insights into ecosystem services additional to yield and economic returns. We also do not yet know the social implications of the regenerative system—the ASP system is more difficult to manage than the BAU, which requires managing only two crops per year. The ASP rotation with its five cash crops and two cover crops requires more time and labor management especially at scale. While the ASP system may allow more efficient use of existing resources by more evenly spreading labor requirements over the growing season, it may also introduce decision and management stresses not as prevalent in our BAU system.

Thus far, we have learned that the early management challenges of our ASP system are addressable insofar as the system has been implemented without sacrificing overall profitability. We attribute early success to properly sequencing entry points and closely tailoring input costs to needs.

## 5 CONCLUSIONS

Our results suggest that US Upper Midwest corn–soybean rotations that are diversified to include additional grain and forage crops can be profitable during the transition years, although economic returns for any individual crop will vary in comparison to the more stable returns of BAU corn and soybean crops. That said, whole rotation comparisons of similar entry points over the 3 years of this study show equivalent economic returns for BAU and ASP, despite occasional yield differences, owing largely to the ASP system's reduced operational costs. That profit gaps tended to close over the course of our study suggests a positive trajectory toward greater future ASP success and reflects the challenges associated with transitioning to a new and more complex cropping system. Knowledge of specific challenges provides opportunities to develop mitigation strategies for future transitions.

## AUTHOR CONTRIBUTIONS

**Ashim Datta**: Formal analysis; investigation; methodology; writing—original draft; writing—review and editing. **Brook Wilke**: Conceptualization; formal analysis; methodology; writing—original draft; writing—review and editing. **Christine Charles**: Conceptualization; formal analysis; methodology; writing—review and editing. **Marc Hasenick**: Conceptualization; formal analysis; methodology. **Tayler Ulbrich**: Conceptualization; methodology; writing—review and editing. **Maninder Singh**: Conceptualization; writing—review and editing. **Molly Sears**: Methodology; validation; writing—review and editing. **G. Philip Robertson**: Conceptualization; project administration; resources; supervision; writing—original draft; writing—review and editing.

## CONFLICT OF INTEREST STATEMENT

The authors declare no conflicts of interest.

## Supporting information




**Supplemental Material**: Supplemental material includes summaries of agronomic management and experiment maps.

## Data Availability

Data are available at Dryad (https://doi.org/10.5061/dryad.kwh70rzj3).

## References

[jeq270084-bib-0001] Baffaut, C. , Metz, M. , Moriasi, D. , Malone, R. , Witthaus, L. , Wacha, K. , Goslee, S. , Hsieh, H.‐Y. , & Robertson, G. P. (2025). Are historical trends in weather consistent with model predictions in the Central United States? Journal of Environmental Quality, 1‐16. 10.1002/jeq2.70066 PMC12593264 40770378

[jeq270084-bib-0002] Bergtold, J. S. , Ramsey, S. , Maddy, L. , & Williams, J. R. (2017). A review of economic considerations for cover crops as a conservation practice. Renewable Agriculture and Food Systems, 34, 62–76. 10.1017/S1742170517000278

[jeq270084-bib-0003] Bowles, T. M. , Mooshammer, M. , Socolar, Y. , Calderón, F. , Cavigelli, M. A. , Culman, S. W. , Deen, W. , Drury, C. F. , Garcia y Garcia, A. , Gaudin, A. C. M. , Harkrom, W. S. , Lehman, R. M. , Osborne, S. L. , Robertson, G. P. , Salerno, J. , Schmer, M. R. , Strock, J. , & Grandy, A. S. (2020). Long‐term evidence shows that crop‐rotation diversification increases agricultural resilience to adverse growing conditions in North America. One Earth, 2, 284–293. 10.1016/j.oneear.2020.02.007

[jeq270084-bib-0004] Bybee‐Finley, K. A. , Muller, K. , White, K. E. , Cavigelli, M. A. , Han, E. , Schomberg, H. H. , Snapp, S. , Viens, F. , Correndo, A. A. , Deiss, L. , Fonteyne, S. , Garcia y Garcia, A. , Gaudin, A. C. M. , Hooker, D. C. , Janovicek, K. , Jin, V. , Johnson, G. , Karsten, H. , Liebman, M. , … Bowles, T. M. (2024). Rotational complexity increases cropping system output under poorer growing conditions. One Earth, 7, 1638–1654. 10.1016/j.oneear.2024.07.008

[jeq270084-bib-0005] Chahal, I. , Vyn, R. J. , Mayers, D. , & Van Eerd, L. L. (2020). Cumulative impact of cover crops on soil carbon sequestration and profitability in a temperate humid climate. Scientific Reports, 10, 13381. 10.1038/s41598-020-70224-6 32770008 PMC7414211

[jeq270084-bib-0006] Che, Y. , Rejesus, R. M. , Cavigelli, M. A. , White, K. E. , Aglasan, S. , Knight, L. G. , Dell, C. , Hollinger, D. , & Lane, E. D. (2023). Long‐term economic impacts of no‐till adoption. Soil Security, 13, 100103. 10.1016/j.soisec.2023.100103

[jeq270084-bib-0007] Crookston, R. K. , Kurle, J. E. , Copeland, P. J. , Ford, J. H. , & Lueschen, W. E. (1991). Rotational cropping sequence affects yield of corn and soybean. Agronomy Journal, 83, 108–113. 10.2134/agronj1991.00021962008300010026x

[jeq270084-bib-0008] Cusser, S. , Bahlai, C. , Swinton, S. M. , Robertson, G. P. , & Haddad, N. M. (2020). Long‐term research avoids spurious and misleading trends in sustainability attributes of no‐till. Global Change Biology, 26, 3715–3725. 10.1111/gcb.15080 32175629

[jeq270084-bib-0009] DeFelice, M. S. , Carter, P. R. , & Mitchell, S. B. (2006). Influence of tillage on corn and soybean yield in the United States and Canada. Crop Management, 5, 1–17. 10.1094/CM-2006-0626-01-RS

[jeq270084-bib-0010] Delgado, J. A. , D'Adamo, R. E. , Villacis, A. H. , Halvorson, A. D. , Stewart, C. E. , Floyd, B. A. , Del Grosso, S. J. , Manter, D. K. , & Alwang, J. (2024). Long‐term effects of nitrogen and tillage on yields and nitrogen use efficiency in irrigated corn. Agronomy, 14, 2304. 10.3390/agronomy14102304

[jeq270084-bib-0011] Garbelini, L. G. , Franchini, J. C. , Debiasi, H. , Balbinot, A. A. Jr. , Betioli, E. Jr. , & Telles, T. S. (2020). Profitability of soybean production models with diversified crops in the autumn–winter. Agronomy Journal, 112, 4092–4103. 10.1002/agj2.20308

[jeq270084-bib-0012] Gathala, M. K. , Kumar, V. , Sharma, P. C. , Saharawat, Y. S. , Jat, H. S. , Singh, M. , Kumar, A. , Jat, M. L. , Humphreys, E. , Sharma, D. K. , Sharma, S. , & Ladha, J. K. (2013). Optimizing intensive cereal‐based cropping systems addressing current and future drivers of agricultural change in the northwestern Indo‐Gangetic Plains of India. Agriculture, Ecosystems & Environment, 177, 85–97. 10.1016/j.agee.2013.06.002

[jeq270084-bib-0013] Giller, K. E. , Hijbeek, R. , Andersson, J. A. , & Sumberg, J. (2021). Regenerative agriculture: An agronomic perspective. Outlook on Agriculture, 50, 13–25. 10.1177/0030727021998063 33867585 PMC8023280

[jeq270084-bib-0014] Gomez, K. A. , & Gomez, A. A. (1984). Statistical procedures for agricultural research. Wiley.

[jeq270084-bib-0015] Guo, T. , Marquart‐Pyatt, S. , & Robertson, G. P. (2025). Building ties at multi‐stakeholder engagement events to facilitate social learning about contentious issues in natural resource management. Agriculture and Human Values, 42, 983–996. 10.1007/s10460-024-10648-2

[jeq270084-bib-0016] Guo, T. , Marquart‐Pyatt, S. T. , & Robertson, G. P. (2023). Using three consecutive years of farmer survey data to identify prevailing conservation practices in four Midwestern US states. Renewable Agriculture and Food Systems, 38, E44. 10.1017/S1742170523000364

[jeq270084-bib-0017] Hamilton, S. K. (2015). Water quality and movement in agricultural landscapes. In S. K. Hamilton , J. E. Doll , & G. P. Robertson (Eds.), The ecology of agricultural landscapes: Long‐term research on the path to sustainability (pp. 275–309). Oxford University Press.

[jeq270084-bib-0018] Hamilton, S. K. , Hussain, M. Z. , Lowrie, C. , Basso, B. , & Robertson, G. P. (2018). Evapotranspiration is resilient in the face of land cover and climate change in a humid temperate catchment. Hydrological Processes, 32, 655–663. 10.1002/hyp.11447

[jeq270084-bib-0019] Hsieh, H.‐Y. , Bohm, S. , & Robertson, G. P. (in press). Kellogg Biological Station climate trends . Zenodo. 10.5281/zenodo.11037062

[jeq270084-bib-0020] Khangura, R. , Ferris, D. , Wagg, C. , & Bowyer, J. (2023). Regenerative agriculture—A literature review on the practices and mechanisms used to improve soil health. Sustainability, 15, 2338. 10.3390/su15032338

[jeq270084-bib-0021] Kleinman, P. J. A. , Spiegal, S. , Rigby, J. R. , Goslee, S. C. , Baker, J. M. , Bestelmeyer, B. T. , Boughton, R. K. , Bryant, R. B. , Cavigelli, M. A. , Derner, J. D. , Duncan, E. W. , Goodrich, D. C. , Huggins, D. R. , King, K. W. , Liebig, M. A. , Locke, M. A. , Mirsky, S. B. , Moglen, G. E. , Moorman, T. B. , … Walthall, C. L. (2018). Advancing the sustainability of US agriculture through long‐term research. Journal of Environmental Quality, 47, 1412–1425. 10.2134/jeq2018.05.0171 30512071

[jeq270084-bib-0022] LaCanne, C. E. , & Lundgren, J. G. (2018). Regenerative agriculture: Merging farming and natural resource conservation profitably. PeerJ, 6, e4428. 10.7717/peerj.4428 29503771 PMC5831153

[jeq270084-bib-0023] Liebig, M. A. , Abendroth, L. J. , Robertson, G. P. , Augustine, D. , Boughton, E. H. , Bagley, G. , Busch, D. L. , Clark, P. , Coffin, A. W. , Dalzell, B. J. , Dell, C. J. , Fortuna, A. , Freidenreich, A. , Heilman, P. , Helseth, C. , Huggins, D. R. , Johnson, J. M. F. , Khorchani, M. , King, K. , … Yost, J. (2024). The LTAR Common Experiment: Facilitating improved agricultural sustainability through coordinated cross‐site research. Journal of Environmental Quality, 53, 787–801. 10.1002/jeq2.20636 39406692

[jeq270084-bib-0024] Miller, S. , Clark, C. , Rutledge, Z. , & Colella, F. (2023). 2023 Michigan State University custom work rates . M. M. S. U. Extension. https://www.canr.msu.edu/farm_management/uploads/files/2023%20MSU%20Custom%20Work%20Rates.pdf

[jeq270084-bib-0025] Morrison, M. J. , Cober, E. R. , Gregorich, E. G. , Voldeng, H. D. , Ma, B. , & Topp, G. C. (2018). Tillage and crop rotation effects on the yield of corn, soybean, and wheat in eastern Canada. Canadian Journal of Plant Science, 98, 183–191. 10.1139/cjps-2016-0407

[jeq270084-bib-0026] National Climatic Data Center . (2013). Summary of monthly normals 1981–2010 . Gull Lake Biology Station. www.ncdc.noaa.gov/cdo‐web/search

[jeq270084-bib-0027] Pedersen, P. , & Lauer, J. G. (2003). Corn and soybean response to rotation sequence, row spacing, and tillage system. Agronomy Journal, 95, 965–971. 10.2134/agronj2003.9650

[jeq270084-bib-0028] Pittelkow, C. M. , Liang, X. , Linquist, B. A. , van Groenigen, K. J. , Lee, J. , Lundy, M. E. , van Gestel, N. , Six, J. , Venterea, R. T. , & van Kessel, C. (2015). Productivity limits and potentials of the principles of conservation agriculture. Nature, 517, 365–368. 10.1038/nature13809 25337882

[jeq270084-bib-0029] Pittelkow, C. M. , Linquist, B. A. , Lundy, M. E. , Liang, X. , Van Groenigen, K. J. , Lee, J. , Van Gestel, N. , Six, J. , Venterea, R. T. , & Van Kessel, C. (2015). When does no‐till yield more? A global meta‐analysis. Field Crops Research, 183, 156–168. 10.1016/j.fcr.2015.07.020

[jeq270084-bib-0030] Pratt, M. R. , Tyner, W. E. , Muth, D. J. , & Kladivko, E. J. (2014). Synergies between cover crops and corn stover removal. Agricultural Systems, 130, 67–76. 10.1016/j.agsy.2014.06.008

[jeq270084-bib-0031] Rasmussen, P. E. , Goulding, K. W. T. , Brown, J. R. , Grace, P. R. , Janzen, H. H. , & Korschens, M. (1998). Long‐term agroecosystem experiments: Assessing agricultural sustainability and global change. Science, 282, 893–896. 10.1126/science.282.5390.893 9794751

[jeq270084-bib-0032] Robertson, G. P. , Allen, V. G. , Boody, G. , Boose, E. R. , Creamer, N. G. , Drinkwater, L. E. , Gosz, J. R. , Lynch, L. , Havlin, J. L. , Jackson, L. E. , Pickett, S. T. A. , Pitelka, L. , Randall, A. , Reed, A. S. , Seastedt, T. R. , Waide, R. B. , & Wall, D. H. (2008). Long‐term agricultural research: A research, education, and extension imperative. Bioscience, 58, 640–645. 10.1641/B580711

[jeq270084-bib-0033] Robertson, G. P. , Gross, K. L. , Hamilton, S. K. , Landis, D. A. , Schmidt, T. M. , Snapp, S. S. , & Swinton, S. M. (2014). Farming for ecosystem services: An ecological approach to production agriculture. Bioscience, 64, 404–415. 10.1093/biosci/biu037 26955069 PMC4776676

[jeq270084-bib-0034] Robertson, G. P. , Wilke, B. , Ulbrich, T. C. , Haddad, N. , Hamilton, S. K. , Baas, D. G. , Basso, B. , Blesh, J. , Boring, T. J. , Campbell, L. , Cassida, K. , Charles, C. , Chen, J. , Doll, J. , Guo, T. , Kravchenko, A. N. , Landis, D. A. , Marquart‐Pyatt, S. T. , Singh, M. , … Stegink, J. (2024). The LTAR cropland common experiment at the Kellogg Biological Station. Journal of Environmental Quality, 53, 893–903. 10.1002/jeq2.20638 39414563

[jeq270084-bib-0035] Roth, R. T. , Ruffatti, M. D. , O'Rourke, P. D. , & Armstrong, S. D. (2018). A cost analysis approach to valuing cover crop environmental and nitrogen cycling benefits: A central Illinois on farm case study. Agricultural Systems, 159, 69–77. 10.1016/j.agsy.2017.10.007

[jeq270084-bib-0036] Sijtsma, C. H. , Campbell, A. J. , McLaughlin, N. B. , & Carter, M. R. (1998). Comparative tillage costs for crop rotations utilizing minimum tillage on a farm scale. Soil and Tillage Research, 49, 223–231. 10.1016/S0167-1987(98)00175-5

[jeq270084-bib-0037] Spiegal, S. , Bestelmeyer, B. T. , Archer, D. W. , Augustine, D. J. , Boughton, E. H. , Boughton, R. K. , Cavigelli, M. A. , Clark, P. E. , Derner, J. D. , Duncan, E. W. , Hapeman, C. , Harmel, D. H. , Heilman, P. , Holly, M. A. , Huggins, D. R. , King, K. , Kleinman, P. J. A. , Liebig, M. A. , Locke, M. A. , … Walthall, C. L. (2018). Evaluating strategies for sustainable intensification of US agriculture through the Long‐Term Agroecosystem Research network. Environmental Research Letters, 13, 034031. 10.1088/1748-9326/aaa779

[jeq270084-bib-0038] Swinton, S. M. , Lupi, F. , Robertson, G. P. , & Landis, D. A. (2006). Ecosystem services from agriculture: Looking beyond the usual suspects. American Journal of Agricultural Economics, 88, 1160–1166. 10.1111/j.1467-8276.2006.00927.x

[jeq270084-bib-0039] USDA Risk Management Agency (RMA) . (2024). Price discovery tool . https://public‐rma.fpac.usda.gov/apps/PriceDiscovery/GetPrices/YourPrice

[jeq270084-bib-0040] Vendig, I. , Guzman, A. , De La Cerda, G. , Esquivel, K. , Mayer, A. C. , Ponisio, L. , & Bowles, T. M. (2023). Quantifying direct yield benefits of soil carbon increases from cover cropping. Nature Sustainability, 6, 1125–1134. 10.1038/s41893-023-01131-7

[jeq270084-bib-0041] Volsi, B. , Higashi, G. E. , Bordin, I. , & Telles, T. S. (2022). The diversification of species in crop rotation increases the profitability of grain production systems. Scientific Reports, 12, 19849. 10.1038/s41598-022-23718-4 36400822 PMC9674645

[jeq270084-bib-0042] Wang, S. , Di Tommaso, S. , Deines, J. M. , & Lobell, D. B. (2020). Mapping twenty years of corn and soybean across the US Midwest using the Landsat archive. Scientific Data, 7, 307. 10.1038/s41597-020-00646-4 32934216 PMC7493954

[jeq270084-bib-0043] Whalen, J. K. , Prasher, S. O. , & Benslim, H. (2007). Monitoring corn and soybean agroecosystems after establishing no‐tillage practices in Québec, Canada. Canadian Journal of Plant Science, 87, 841–849. 10.4141/CJPS06023

[jeq270084-bib-0044] Yang, X. , Xiong, J. , Du, T. , Ju, X. , Gan, Y. , Li, S. , Xia, L. , Shen, Y. , Pacenka, S. , Steenhuis, T. S. , Siddique, K. H. M. , Kang, S. , & Butterbach‐Bahl, K. (2024). Diversifying crop rotation increases food production, reduces net greenhouse gas emissions and improves soil health. Nature Communications, 15, 198. 10.1038/s41467-023-44464-9 PMC10764956 38172570

