# Supplementary material for: Crop performance and profitability for the initial transition years of a regenerative cropping system in the Upper Midwest United States
Source: J Environ Qual. 2025 Sep 23;54(6):1572–85. doi: 10.1002/jeq2.70084 (PMC12593274; doi:10.1002/jeq2.70084)
Supplement: Supplementary file 1 — Supplemental Material: Supplemental material includes summaries of agronomic management and experiment maps. [file JEQ2-54-1572-s001.docx]

**Supplemental Material**

**Crop performance and profitability for the initial transition years of a regenerative cropping system in the Upper Midwest USA**

Ashim Datta^1,2,3,4^, Brook Wilke*^1^, Christine Charles^1^, Marc Hasenick^5^, Tayler Ulbrich^1^, Maninder Singh^2^, Molly Sears^6^, G.Philip Robertson^1,2,3^

^1^W. K. Kellogg Biological Station, Michigan State University, Hickory Corners, MI 49060, USA

^2^Department of Plant, Soil and Microbial Sciences, Michigan State University, East Lansing, MI 48824, USA

^3^Great Lakes Bioenergy Research Center, Michigan State University, East Lansing, MI 48824, USA

^4^Division of Soil and Crop Management, ICAR-Central Soil Salinity Research Institute, Karnal, Haryana 132001, India

^5^Hasenick Farms, Springport, MI 49284

^6^Department of Agricultural, Food, and Resource Economics, Michigan State University, East Lansing, MI 48824, USA

*Corresponding author: wilkebro@msu.edu

**Contents**

1. Soil properties of the field site.
2. Table S1. Field operations and crop management details for corn, soybean, cover crop, wheat, canola and forage under Business as usual and Aspirational cropping systems in 2022.
3. Table S2. Field operations and crop management details for corn, soybean, cover crop, wheat, canola and forage under Business as usual and Aspirational cropping systems in 2023.
4. Table S3. Field operations and crop management details for corn, soybean, cover crop, wheat, canola and forage under Business as usual and Aspirational cropping systems in 2024.
5. Figure S1. Layout of the KBS Aspirational Cropping System Experiment plots.
6. Figure S2. Weather patterns for the experiment years a) 2022, b) 2023 and c) 2024.
7. References

**Soil properties of the field site**

Soils at the site formed from glacial outwash and loess from regional outwash plains following retreat of the Wisconsin glaciation ~18,000 years ago. Predominant soils around KBS are well-drained Alfisols; KBS LTAR experiments are under Typic Hapludalfs, co-mingled Kalamazoo and Oshtemo series loams and sandy loams (Crum & Collins, 1995) with intermixed loess (Luehmann et al., 2016). Soils developed on a fairly level outwash plain with highly permeable soils, such that the main pathway of water movement off the fields besides evapotranspiration is infiltration and percolation to a water table that lies ~15 m beneath the surface, although runoff can occur during heavy storms.

Soil samples were collected from 0–25 cm soil depths using a 2 cm diameter probe before the initiation of the experiment in December, 2021. Plots were sampled near three permanent sampling stations within each plot, and fields were sampled on a 2.5 acre grid. Soil samples were air-dried, ground to pass a 4-mm sieve, then stored in plastic containers for analysis of selected soil chemical properties by a commercial soil analysis laboratory (A&L Great Lakes, Fort Wayne, IN). Soil pH was measured in a 1:1 soil:water ratio solution. Available P, K, Ca, and Mg (Mehlich III extraction) were measured by Inductively Couple Plasma Atomic Emission Spectroscopy (ICP AES). Initial soil properties of the experimental site were: soil pH = 6.14 ±0.12 (SD, n=23), available P = 48.6 ±5.1 µg/g soil (n=23), K = 124±11.3 µg/g (n=23), Ca = 968±45.5 µg/g, Mg = 182±11 µg/g (n=23), and CEC = 7.8±0.5 me/100g (n=23).

**Table S1.** Field operations and crop management details for corn, soybean, cover crop, wheat, canola, and forage in different rotation phases of the Business as usual (BAU) and Aspirational (ASP) cropping systems in 2022.

| Treatment | BAU1 | BAU2 | ASP1 | ASP2 | ASP3 | ASP4 | ASP5 |
| --- | --- | --- | --- | --- | --- | --- | --- |
| Crop | Corn | Soybean | Corn | Soybean | Spring wheat | Spring canola | Perennial forage |
| Previous crop | Corn | Corn | Corn | Corn | Corn | Corn | Corn |
| Variety | Pioneer P0720Q Treated | Asgrow 24XF1 Treated | Dekalb DKC 55-53 Treated | Asgrow 21XF1 Un-Treated | Ryan Soft White Treated | NCC101S Un-treated | Alfalfa, Red Clover, Chicory, Italian Ryegrass, Oats (Innoculated) |
| Date of sowing | 5/13/2022 | 5/10/2022 | 5/13/2022 | 5/10/2022 | 4/11/2022 | 4/12/2022 | 4//13/2022 |
| Row spacing | 76.2 cm | 19.05 cm | 76.2 cm | 19.05 cm | 19.05 cm | 19.05 cm | 19.05 cm |
| Seeding rate | 74,100 seeds/ha | 358,150 seeds/ha | 69,160 seeds/ha  (Variable Rate Fields) | 395,200 seeds/ha | 3,211,000  seeds/ha | 358,150  seeds/ha | 17.93 kg/ha Perennials, 33.6 kg/ha Oats |
| Tillage | 4/29/22- Chisel Plowed 5/11/22 - Soil Finished | 4/29/22- Chisel Plowed 5/9/22 - Soil Finished | -- | -- | -- | -- | -- |
| Manure/compost | -- | -- | 17.93 Mg/ha Composted Pen Pack Manure (70% Moisture, 0.55% N) | -- | -- | -- | -- |
| Nutrient management | 246.5 kg N/ha, 78.42 kg P_2_O_5_/ha  44.8 kg K_2_O/ha, 29.12 kg S/ha,  0.28 kg Zinc/ha, 0.15 kg Boron/ha | 25.76 kg N/ha, 44.81 kg P_2_O_5_/ha  84.03 kg K_2_O/ha, 25.76 kg S/ha, | 168.06 kg N/ha, 16.8 kg P_2_O_5_/ha  6.69 kg S/ha  0.28 kg Zinc/ha, 0.15 kg Boron/ha | 12.33 kg N/ha,  67.23 kg K_2_O/ha, 13.44 kg S/ha | 95.24 kg N/ha, 21.29 kg P_2_O_5_/ha  67.23 kg K_2_O/ha, 16.8 kg S/ha | 84.03 kg N/ha, 21.29 kg P_2_O_5_/ha  67.23 kg K_2_O/ha, 16.8 kg S/ha | 78.42 kg N/ha,  134.44 kg K_2_O/ha, 6.72 kg S/ha |
| Weed management | Pre-Emerge  Warrant, Sharpen, Glyphosate  Post-Emerge  Armezon Pro, Glyphosate | Pre-Emerge  Zidua Pro, Metribuzin, Sharpen  Post-Emerge  Glyphosate, Glufosinate | Pre-Emerge  Dual II Magnum, Sharpen, Glyphosate  Post-Emerge  Armezon Pro, Glyphosate | Pre-Emerge  Zidua Pro, Metribuzin, Sharpen  Post-Emerge  Glyphosate, Glufosinate | Pre-Emerge  Glyphosate, Glufosinate  Post-Emerge  Harmony Extra | Pre-Emerge  Glyphosate, Glufosinate  Post-Emerge  Stinger | Pre-Emerge  Glyphosate, Glufosinate |
| Water management | Rain water received during May to September 452 mm | Rain water received during May to September 452 mm | Rain water received during May to September 452 mm | Rain water received during May to September 452 mm | Rain water received during May to September 452 mm | Rain water received during May to September 452 mm | Rain water received during May to September 452 mm |
| Pest management | 7/25/22  Miravis Neo Fungicide, Tomstone Helios Insecticide |  | 7/25/22  Miravis Neo Fungicide, |  | 6/13/22  Miravis Ace Fungicide |  |  |
| Date of harvesting | 11//7/2022 | 9/30/2022 | 11/7/2022 | 9/30/2022 | 7/27/2022 | 7/26/2022 | 6/16/22, 7/28/22, 8/30/22 |
| Cover crop |  |  | 6/28/22 Inter-seed Crimson Clover, Radish, Rapeseed  11/9/22 Cereal Rye | 10/4/22  Winter Wheat Planting | 8/2/22 – 9/14/22  Sorghum-Sudan, Pearl Millet, Sunn Hemp (+Fertilizer, Herbicide) | 8/26/22  Alfalfa, Red Clover, Chicory, Italian Ryegrass |  |

**Table S2.** Field operations and crop management details for corn, soybean, cover crop, wheat, canola, and forage in different rotation phases of the Business as usual (BAU) and Aspirational (ASP) cropping systems in 2023.

| Treatment | BAU1 | BAU2 | ASP1 | ASP2 | ASP3 | ASP4 | ASP5 |
| --- | --- | --- | --- | --- | --- | --- | --- |
| Crop | Soybean | Corn | Soybean | Winter Wheat | Winter Canola | Perennial Forage | Corn |
| Previous crop | Corn | Soybean | Winter Canola | Soybean | Winter Wheat | Winter Canola | Perennial Forage |
| Variety | Asgrow 26XF1 with Insecticide + Fungicide Seed Treatment | Pioneer 0720AM with Insecticide + Fungicide Seed Treatment | Asgrow 21XF1 Untreated | Sunburst with Fungicide Seed Treatment | Plurax CL Untreated | KF 425 HD Alfalfa, KF 257 Alfalfa, KF 401 B Alfalfa, Freedom Red Clover, Cyclone Red Clover, Chicory UT, KF Allegro Annual Ryegrass | Pioneer P0035AM with Insecticide + Fungicide Seed Treatment |
| Date of sowing | 4/13/2023 | 5/5/2023 | 5/21/2023 | 10/4/2022 | 9/21/2022  Replanted spring canola in some areas on 4/11/23 | 8/29/2022 | 5/12/2023 |
| Row spacing | 19.05 cm | 76.2 cm | 19.05 cm | 19.05 cm | 38.1 cm | 19.05 cm | 76.2 cm |
| Seeding rate | 3,76,613 seeds/ha | 74,100 seeds/ha | 3,21,100 seeds/ha | 33,34,500 seeds/ha | 6,54,550 seeds/ha | 6.72 kg Alfalfa, 4.47 kg red clover, 4.47 kg ryegrass and 1.11 kg chicory/ha | 69,160 seeds/ha  (Variable Rate Fields) |
| Tillage | 12/8/22- Chisel Plowed 4/13/23 - Soil Finished | 12/8/22- Chisel Plowed 5/5/22 - Soil Finished | -- | -- | -- | -- | -- |
| Manure/compost | -- | -- | -- | -- | -- | -- | 22.42 Mg/ha Composted Pen Pack Manure (76% Moisture, 0.41% N) |
| Nutrient management | 29.13 kg N/ha, 17.93 kg P_2_O_5_/ha,  84.03 kg K_2_O/ha, 31.37 kg S/ha, | 224 kg N/ha, 78.42 kg P_2_O_5_/ha,  50.41 kg K_2_O/ha, 51.55 kg S/ha,  0.28 kg Zinc/ha, 0.22 kg Boron/ha, 0.21 kg /ha Titan XC Micronutrients | 23.54 kg N/ha, 67.23 kg K_2_O/ha, 26.89 kg S/ha | 134.44 kg N/ha, 33.62 kg P_2_O_5_/ha,  67.22 kg K_2_O/ha, 30.25 kg S/ha, 0.08 kg Boron/ha | 158 kg N/ha, 33.61 kg P_2_O_5_/ha,  67.22 kg K_2_O/ha, 31.37 kg S/ha | 75.06 kg N/ha, 67.23 kg K_2_O/ha, 26.9 kg S/ha | 100.83 kg N/ha, 18.48 kg S/ha, 0.28 kg Zinc/ha |
| Weed management | Post-Emerge (2 Applications)  5/9/23 -Warrant, Glufosinate  6/16/23 - Glyphosate | Post-Emerge  5/23/23 – Glyphosate, Armezon pro, Warrant | Pre-Emerge  Glufosinate, Metribuzin, Sharpen, Warrant  Post-Emerge  Glyphosate | Pre-Emerge  Glyphosate (Fields Only)  Post-Emerge  Huskie | Pre-Emerge  Glyphosate  Post-Emerge  Beyond | Pre-Emerge  Glyphosate, Glufosinate | Pre-Emerge  5/4/23 – Metolachlor, 2,4-D, Dicamba, Glyphosate  Post-Emerge  7/5/23 - Glyphosate |
| Water management | Rain water received during May to September 307 mm | Rain water received during May to September 307mm | Rain water received during May to September 307 mm | Rain water received during May to September 307 mm | Rain water received during May to September 307 mm | Rain water received during May to September 307 mm | Rain water received during May to September 307 mm |
| Pest management |  | 8/7/23 – Miravis Neo Fungicide, Tombstone Helios Insecticide |  |  |  |  |  |
| Date of harvesting | 10/3/ 2023 | 10/27/2023 | 10/11/2023 | Grain – 7/12/23, Straw – 7/14/23 | 7/24/2023 | 5/15/23, 6/19/23, 7/31/23, 8/25/23 | 11/16/23 |
| Cover crop |  |  | 10/11/23 Winter Wheat Planting | 7/14/23 – Planted Sudex, Pearl Millet, Sunn Hemp, which was fertilized with 43 lbs N/A and 7 lbs S/A and harvested on 9/1/23 for forage  9/14/23 – Winter Canola Planting | 8/16/23  Alfalfa, Red Clover, Chicory, Italian Ryegrass |  | 7/10/23 – Dwarf Essex Rape, Tillage Radish, Crimson Clover  11/15/23 – Cereal Rye |

**Table S3.** Field operations and crop management details for corn, soybean, cover crop, wheat, canola, and forage in different rotation phases of the Business as usual (BAU) and Aspirational (ASP) cropping systems in 2024.

| Treatment | BAU1 | BAU2 | ASP1 | ASP2 | ASP3 | ASP4 | ASP5 |
| --- | --- | --- | --- | --- | --- | --- | --- |
| Crop | Corn | Soybean | Winter Wheat | Winter Canola | Perennial Forage | Corn | Soybean |
| Previous crop | Soybean | Corn | Soybean | Winter Wheat | Winter Canola | Perennial Forage | Corn |
| Variety | Dekalb 59-82 RIB with Insecticide + Fungicide Seed Treatment | Asgrow 25XF3 with Insecticide + Fungicide Seed Treatment | Sunburst with Fungicide Seed Treatment | Plurax CL with Fungicide Seed Treatment | KF 425 HD Alfalfa, KF 257 Alfalfa, KF 401 B Alfalfa, Clofford Red Clover, Red Power Red Clover, Six Point Chicory, KF Allegro Annual Ryegrass | Dekalb DKC 101-35 RIB with Insecticide + Fungicide Seed Treatment | Asgrow 20XF4 Untreated |
| Date of sowing | 5/7/24 | 4/25/24 | 10/11/23 | 9/14/23 | 8/16/23 | 5/7/24 | 4/26/24 Replant 5/16/24 and partial replant 6/14/24 |
| Row spacing | 76.2 cm | 19.05 cm | 19.05 cm | 25.4 cm | 19.05 cm | 76.2 cm | 76.2 cm |
| Seeding rate | 79,040 seeds/ha | 8,54,126 seeds/ha | 32,11,000 seeds/ha | 580,450 seeds/ha | 6.72 kg Alfalfa, 4.47 kg red clover, 4.47 kg ryegrass and 1.11 kg chicory/ha | 170,825 seeds/ha (Variable Rate Fields) | 296,400 seeds/ha, 80,000 seeds/ha first replant, 296,400 seeds/ha second partial replant |
| Tillage | 12/8/23- Chisel Plowed 5/2/24 - Soil Finished | 12/8/23- Chisel Plowed 4/25/24 - Soil Finished | -- | -- | -- | -- | -- |
| Manure/compost | -- | -- | -- | -- | -- | 33.16 Mg/ha Composted Pen Pack Manure (76% Moisture, 0.55% N) |  |
| Nutrient management | 224.07 kg N/ha, 156.85 kg K_2_O/ha, 38.09 kg S/ha, 61.62 kg P_2_O_5_/ha, 0.28 kg Zinc/ha | 13.44 kg N/ha, 162.45 lb K_2_O/A, 13.44 kg S/A, 44.81 kg P_2_O_5_/A | 134.44 kg N/ha, 67.22 lb K_2_O/ha, 33.61 kg S/ha, 35.85 kg P_2_O_5_/ha, 0.09 kg Boron/ha | 117.64 kg N/ha, 67.22 kg K_2_O/ha, 33.61 kg S/ha, 35.85 kg P_2_O_5_/ha | 33.61 kg N/ha, 271.13 kg K_2_O/ha, 16.8 kg S/ha | 100.83 kg N/ha, 67.22 kg K_2_O/ha, 13.44 kg S/ha, 17.93 kg P_2_O_5_/ha, 0.28 kg Zinc/ha | 119.88 kg K_2_O/ha |
| Weed management | Post-Emerge  Glyphosate, Armezon pro, Warrant | Pre-Emerge Glyphosate, Warrant, Metribuzin; Post-Emerge Glyphosate, Glufosinate | Post-Emerge  Huskie | Pre-Emerge  Glyphosate, Glufosinate  Post-Emerge  Beyond | Pre-Emerge  Glyphosate, Glufosinate | Pre-Emerge  Glyphosate, Sharpen, 2,4-D, Dicamba;  Post-Emerge  Glyphosate, Armezon pro, Warrant | Pre-Emerge Glyphosate, Warrant, Metribuzin; Post-Emerge Glyphosate, Glufosinate |
| Pest management | R1 Miravis Neo Fungicide &Tombstone Helios Insecticide |  | Flowering Miravis Ace Fungicide |  |  | R1 Miravis Neo Fungicide |  |
| Date of harvesting | 10/24/24 | 9/27/24 | Grain 7/2/24, Straw 7/3/24 | 7/2/24 | 5/17/24, 7/16/24, 9/3/24 | 10/14/24 | 10/3/24 |
| Cover crop |  |  | 7/13/24 Planted Sudex, Pearl Millet, Sunn Hemp, which was fertilized with 50.42 kg N/ha and 7.84 kg S/ha and harvested on 8/30/24 for forage  9/10/24 – Winter Canola Planting |  | 8/14/24  Alfalfa, Red Clover, Chicory, Orchard Grass Planting | 10/18/24 Cereal Rye | 10/4/24 Winter Wheat Planting |


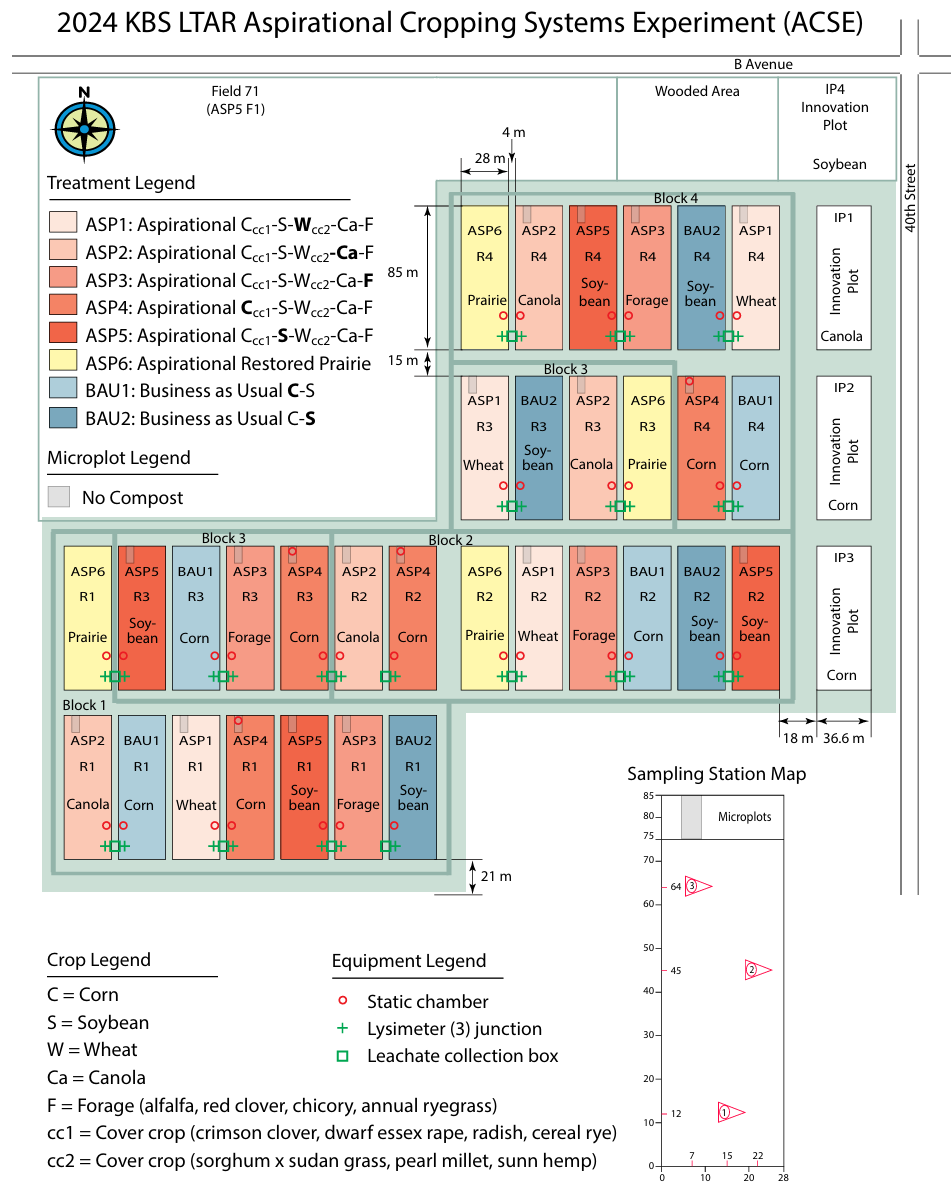


Figure S1. Layout of the plot-scale KBS Aspirational Cropping System Experiment (ACSE).

| a) 2022  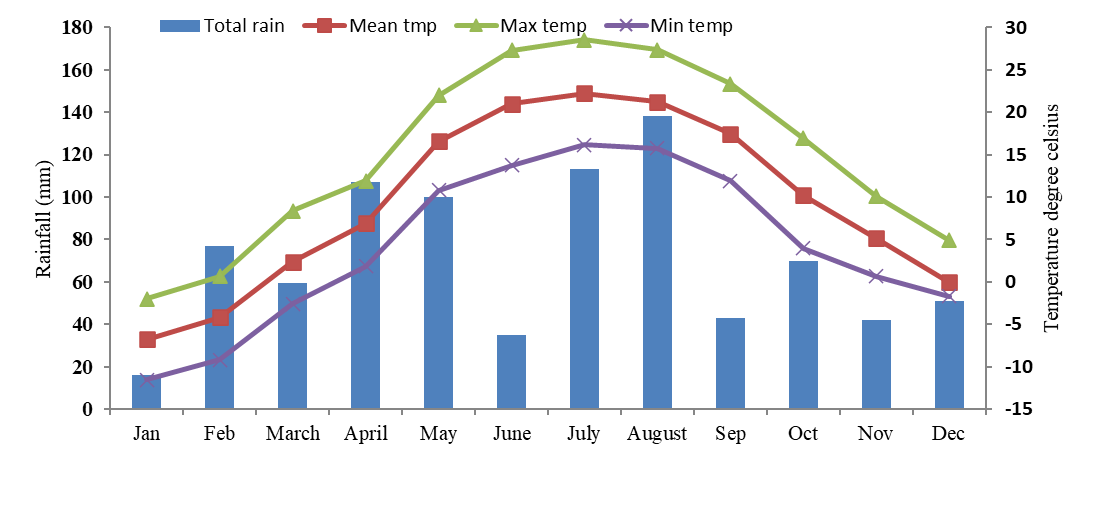 |
| --- |
| b) 2023  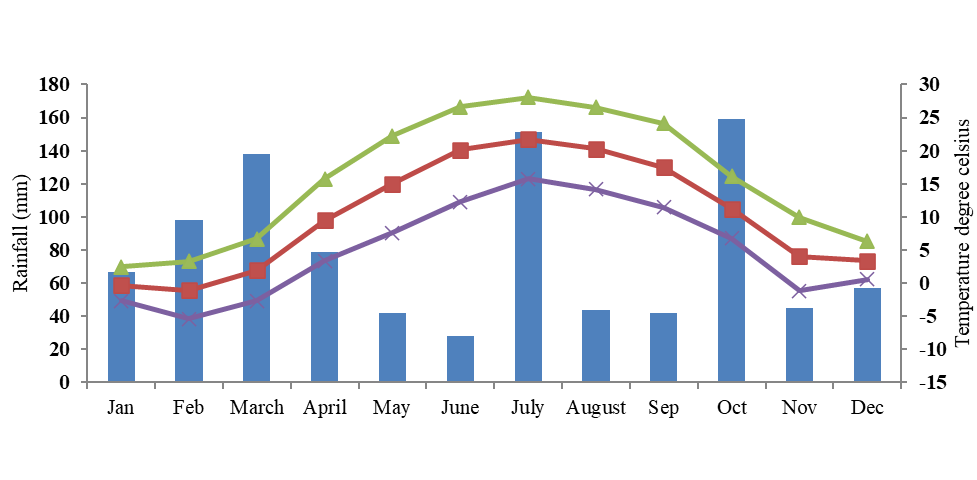 |
| c) 2024  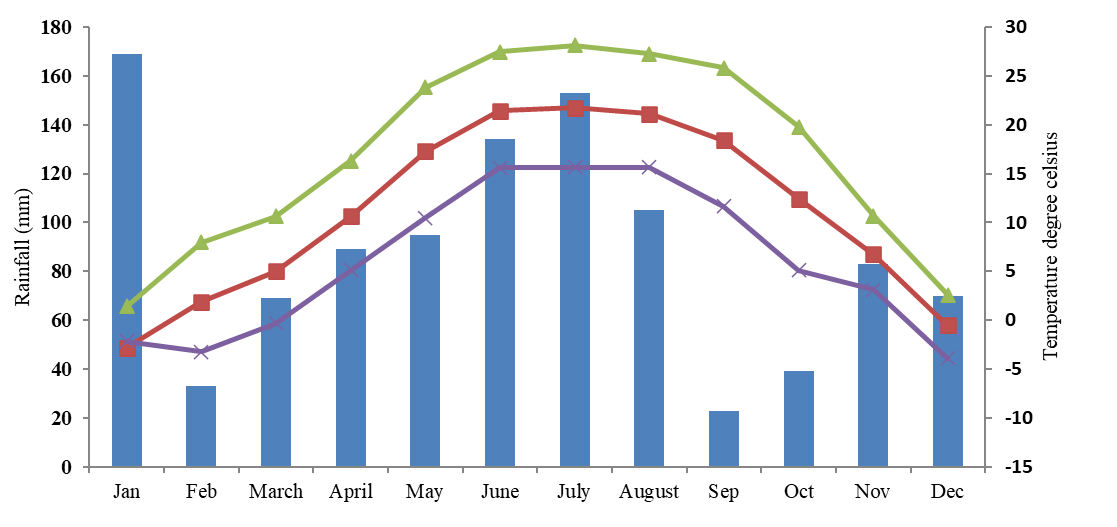 |

Figure S2. Weather patterns for the experiment years a) 2022, b) 2023 and c) 2024.

References

Crum, J.R. & Collins, H.P. (1995). *KBS soils*. Zenodo, <http://doi.org/10.5281/zenodo.2560750>.

Luehmann, M.D., Peter, B.G., Connallon, C.B., Schaetzl, R.J., Smidt, S.J., Liu, W., Kincare, K.A., Walkowiak, T.A., Thorlund, E. & Holler, M.S. (2016). Loamy, two-storied soils on the outwash plains of southwestern lower Michigan: Pedoturbation of loess with the underlying sand. *Annals of the American Association of Geographers*, *106*, 551-572. <http://dx.doi.org/10.1080/00045608.2015.1115388>
